# Supplementary material for: The interactome of intact mitochondria by cross-linking mass spectrometry provides evidence for coexisting respiratory supercomplexes
Source: Mol Cell Proteomics. 2017 Dec 8;17(2):216–32. doi: 10.1074/mcp.RA117.000470 (PMC5795388; doi:10.1074/mcp.RA117.000470)
Supplement: Supplemental Data [file supp_RA117.000470_133922_0_supp_23978_fzffwf.zip › spectra_annotation/mito_spectra_annotation/158-1-2-1-14-1.pdf]

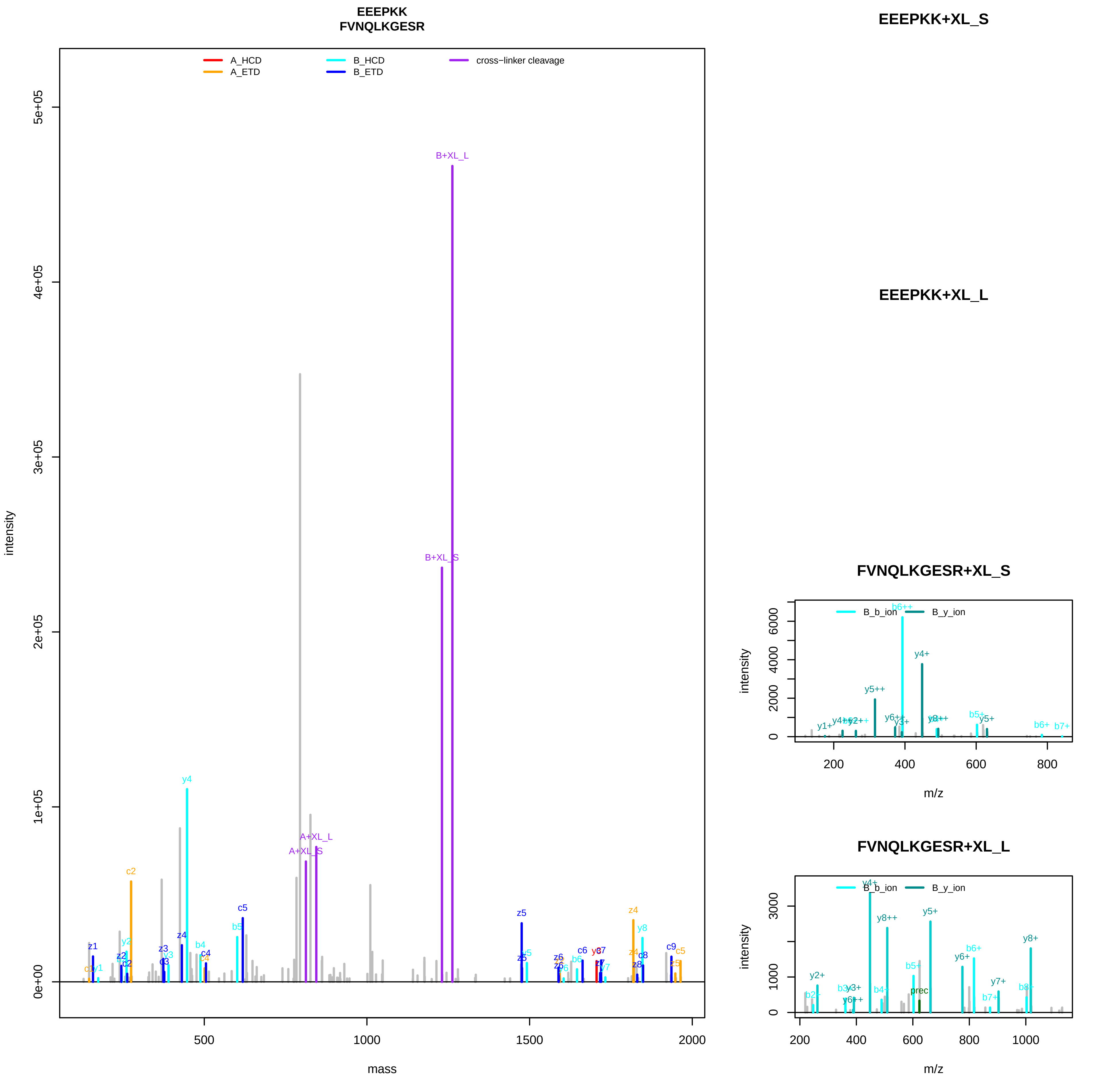
EEEPKK
FVNQLKGESR
A_HCD
A_ETD
B_HCD
B_ETD
cross−linker cleavage
intensity
0e+00
1e+05
2e+05
3e+05
4e+05
5e+05
mass
500
1000
1500
2000
B+XL_L
B+XL_S
A+XL_L
A+XL_S
y4
c2
c5
b5
z5
z4
y8
c9
c5
EEEPKK+XL_S
EEEPKK+XL_L
FVNQLKGESR+XL_S
B_b_ion
B_y_ion
b6++
y4+
y5++
y1+
y6++
y3+
y8++
b5+
y5+
b6+
b7+
m/z
200
400
600
800
0
2000
4000
6000
FVNQLKGESR+XL_L
y4+
y8++
y5+
y2+
b5+
y6+
b6+
y8+
y7+
b7+
b8+
b3+
y3+
b4+
prec
0
1000
2000
3000
1000
